# The Impact of Laser Irradiation on Thin ZrN Films Deposited by Pulsed DC Magnetron Sputtering

**DOI:** 10.3390/nano14241999

**Published:** 2024-12-13

**Authors:** Ameena Nazneen, Penghui Lei, Di Yun

**Affiliations:** School of Nuclear Science and Technology, Xi’an Jiaotong University, Xi’an 710049, China; nazneen.ameena@stu.xjtu.edu.cn

**Keywords:** zirconium nitride, sputtering, voids, thin film, laser

## Abstract

Transition metal nitrides have extensive applications, including magnetic storage devices, hardware resistance coatings, and low-temperature fuel cells. This study investigated the structural, electrical, and mechanical properties of thin zirconium nitride (ZrN) films by examining the effects of laser irradiation times. Thin ZrN films were deposited on glass substrates using pulsed DC magnetron sputtering and irradiated with a diode laser for 6 and 10 min. Characterization was performed using X-ray diffraction (XRD), scanning electron microscopy (SEM), high-resolution transmission electron microscopy (HRTEM), nanoindentation, and four-point probe techniques. Extended laser irradiation times resulted in increased numbers of peaks on XRD analysis, indicating enhanced crystalline behavior of thin ZrN film. SEM analysis revealed surface voids, while HRTEM showed nanostructured ZrN with uniform plane orientation. The electrical properties of the thin ZrN film improved with extended laser irradiation, as demonstrated by a reduction in sheet resistance from 0.43 × 10^9^ Ω to 0.04 × 10^9^ Ω. Additionally, nanoindentation tests revealed an increase in hardness, rising from 8.91 GPa to 9.36 GPa.

## 1. Introduction

In recent years, the demand for high-performance transition metal nitrides (TMNs) in the rock salt phase has been steadily increasing. These TMNs have found diverse applications, including magnetic storage devices, cutting tools, optical coatings, optoelectronic devices, resistance coating for generators, and low-temperature fuel cells. The exceptional properties of TMNs, derived from their mixed ionic, covalent, and metallic bonding, render them unique in amalgamating metallic and non-metallic traits. These properties encompass high melting points, luster, brittleness, high hardness, and high conductivity [1,2,3].

The field of hard-coating metal substrate applications has witnessed substantial growth recently [4]. Among these nitrides, zirconium nitride (ZrN) films have emerged as particularly interesting materials for a wide array of applications, including neutron reactors [5], aerospace components, cryogenic thermometers, diffusion barriers, decorative coatings, and hard coatings. ZrN films are favored for their exceptional corrosion and oxidation resistance [6], superior mechanical properties, lower resistivity, and distinctive warm golden color in comparison to titanium nitride (TiN) [7,8,9,10].

However, the deposition of ZrN films poses specific challenges when compared to materials like TiN and CrN due to zirconium’s higher melting point, lower vapor pressure, and increased susceptibility to oxygen and carbon contamination [11]. The mono-nitrides of fourth-column transition metals exhibit interesting properties resulting from the combination of covalent and metallic bonding characteristics. Covalent crystalline properties contribute to high melting points, exceptional hardness, brittleness, and excellent thermal and chemical inertness, while the metallic attributes include electrical conductivity and metallic reflectance. These materials exhibit a gold-like appearance due to their high reflectance in the red end of the visible spectrum coupled with low reflectance in the ultraviolet region, thus presenting a unique optical signature [12,13,14]. Moreover, zirconium compounds are renowned for their chemical inertness, rendering them suitable for applications such as inert matrix fuel in nuclear reactors [9,15,16,17,18,19].

Physical vapor deposition (PVD) techniques, including RF sputtering, arc evaporation, and ion beam-assisted deposition, have been extensively employed for depositing transition metal nitride films. The properties of zirconium nitride (ZrN) films are significantly influenced by the deposition methods and associated processing parameters [11,20,21,22]. In magnetron sputtering, ion bombardment is widely recognized for its ability to modify film structure, enhancing film properties [23,24,25,26]. Pulsed DC magnetron sputtering has become a promising deposition technology, especially for minimizing arcing events caused by charge buildup on target surfaces. This reduction in arcing helps to maintain stable deposition conditions and prevent the degradation of film properties. The physical characteristics of ZrN films produced via pulsed DC magnetron sputtering are largely influenced by process parameters. For example, researchers studied how substrate temperature affects the tribological properties of thin ZrN films [27,28]. Laser irradiation enhances the surface mobility of condensed species, facilitating the formation of voids, increased crystallinity, and improved the material’s hardness. Additionally, ZrO_2_ can form in thin ZrN films as a result of zirconium reacting with oxygen, driven by oxygen diffusion [29,30,31,32,33,34,35,36,37]. Given that some physical properties of zirconium nitride films are closely related to their microstructure and nitrogen content, this study explored how laser irradiation affects thin ZrN films’ microstructure and electrical and mechanical properties.

In this study, the pulsed DC (direct current) magnetron sputtering technique was chosen for depositing ZrN films onto glass substrates. ZrN coatings, known for their excellent mechanical, decorative, and wear-resistant properties, have been widely used for decades. However, in this work, we aimed to enhance the microstructural, mechanical, and electrical properties of the coatings by incorporating laser irradiation as a post-deposition treatment. Notably, to our knowledge, no prior research has reported the influence on ZrN films using laser surface treatment for 6 and 10 min with this specific laser technique. Our experimental findings suggest that laser irradiation can significantly improve the hardness, crystallinity, and electrical performance of these coatings. This approach has the potential to extend the functional range of thin ZrN films, offering new possibilities for high-performance applications. In this work, the physical, structural, morphological, electrical, and mechanical properties of the films were thoroughly investigated through X-ray diffraction (XRD), scanning electron microscopy (SEM), high-resolution transmission electron microscopy (HRTEM), four-point probe, and nanoindentation techniques.

## 2. Materials and Methods

Thin zirconium nitride (ZrN) films were deposited on glass substrates utilizing a pulsed DC magnetron sputtering system from AJA International, Hingham, MA, USA (AJA ATC 2200) within a vacuum chamber measuring 300 mm in diameter and 350 mm in height. Glass substrates were initially cleaned with a soap solution, then subjected to ultrasonic cleaning in acetone for 15 min, and finally dried with hot air before being placed in a deposition chamber. A high-purity metallic zirconium target (99.99%) with a 3-inch diameter was employed for sputtering. The deposition chamber was evacuated to a base pressure of 2 × 10^−5^ mbar while maintaining the substrate at a constant temperature of 650 K throughout the deposition process. Argon gas (99.99%) served as the sputtering gas, while nitrogen gas (99.99%) acted as the reactive gas. Prior to deposition, a 5 min sputtering process was executed on the zirconium target to eliminate any surface oxide. The flow rates of argon and nitrogen gases were held constant at 40 and 1 sccm, respectively, and the deposition process was conducted for 90 min.

Subsequent to the deposition of thin ZrN films with a thickness of 200 nm, they underwent diode laser irradiation at a visible wavelength of 650 nm for durations of 6 and 10 min. The laser was applied in a defocused beam mode to ensure uniform irradiation across the sample surface. The deposition parameters of the thin ZrN films used in the current investigations are given in Table 1.

These samples were characterized using a variety of techniques. The crystalline structure of the ZrN films was analyzed using X-ray diffraction (XRD) with Cu Kα radiation (λ = 1.5406 Å). XRD patterns were captured at a grazing incidence angle (3°) within the 2θ range of 30°–80°, employing a scan rate of 0.02°. The surface morphology, microstructure, and phase compositional analysis of the thin ZrN films were examined using a scanning electron microscope (Carl Zeiss AG, Oberkochen, Germany) and high-resolution transmission electron microscopy (HRTEM). The HRTEM studies were conducted out on a JEOL 2000 EXII TEM (JEOL, Tokyo, Japan) run at 200 kV. The ZrN films were initially cut into small pieces and thinned to approximately 200 nm using a focused ion beam (FIB) system. After thinning, the samples were cleaned in an ultrasonic bath to remove any surface contaminants. Finally, the thinned films were placed on a suitable support for TEM imaging and analysis. The electrical properties of the thin films were evaluated using the four-point probe technique, while their mechanical properties were measured using nanoindentation techniques. Electrical measurements were performed at room temperature under ultrahigh-vacuum conditions employing a four-tip configuration with a tip spacing of 5 µm, corresponding to a 15 µm separation between the current-carrying probes. Mechanical measurements were performed using the continuous stiffness measurement (CSM) technique on a nanoindenter. The CSM method enabled depth-controlled indentations, with a maximum indentation depth of less than 200 nm. The primary aim of the nanoindentation study was to evaluate the effects of indentation depth on the material’s load, elastic modulus, and hardness. A strain rate of 0.2 s^−1^ was applied during the measurements, and Poisson’s ratio of the sample was determined to be 0.27 at room temperature.

## 3. Results and Discussion

### 3.1. X-Ray Diffraction (XRD)

**Figure 1:**
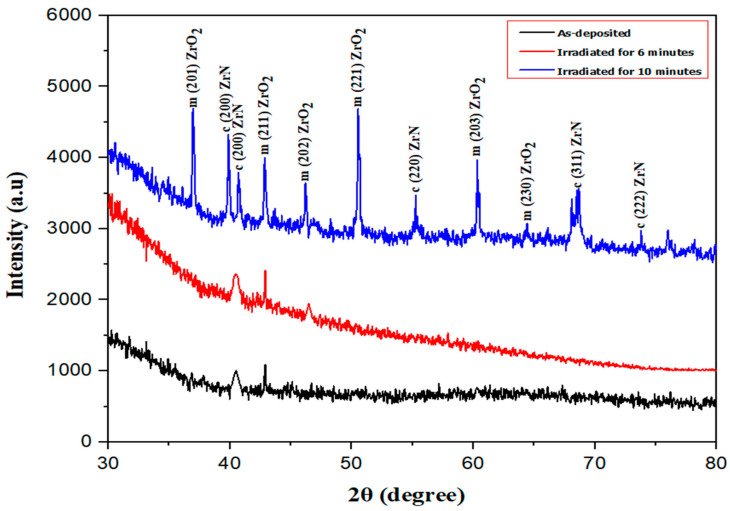
X-ray diffraction pattern of as-deposited/irradiated thin ZrN film. “C” and “m” indicate the cubic and monoclinic planes of ZrN and ZrO_2_.

In Figure 1, the XRD analysis showcases a thin zirconium nitride (ZrN) film deposited on a glass substrate. XRD was used to assess the crystalline nature of the ZrN films. Initially, for as-deposited samples, a single peak of ZrN and ZrO_2_ emerged at 2θ = 40.51° and 42.62°, respectively. Following 6 min of irradiation, one peak of ZrN and two peaks of ZrO_2_ surfaced at 2θ = 40.51°, 42.62°, and 45.90°, respectively. Notably, with 10 min of irradiation, the presence of pronounced ZrN peaks in the diffractogram of the laser-treated surface signified the crystalline behavior of ZrN, showcasing high-intensity peaks [38].

Following a 10-min treatment, a distinct reflection emerged, aligning with the ZrN (2 0 0) orientation at 39.86° and 40.51°. The pristine film’s diffraction patterns reveal two peaks corresponding to stable metallic phases, specifically the cubic ZrN [cF8, Fm-3m], showcasing the (2 0 0) plane reflection at 39.86° and 40.51°. The cubic structure of the zirconium nitride film was identifiable through PDF card 02-0956. Various research groups have documented the simultaneous growth of stable ZrN phases within zirconium nitride films [28,39,40,41,42]. In the case of the sample subjected to a 10-min laser irradiation, novel peaks corresponding to cubic zirconium nitride (ZrN) with planes (2 2 0), (3 1 1), and (2 2 2) were observed at 55.26°, 68.6°, and 73°, respectively [32,43,44,45,46]. Furthermore, peaks aligning with monoclinic ZrO_2_ and its planes (2 0 1), (2 1 1), (2 0 2), (2 2 1), (2 0 3), and (2 3 0) were evident at 36.22°, 42.62°, 45.90°, 50.46°, 60.33°, and 64.27°, respectively [47,48]. The monoclinic structure of the ZrO_2_ film was identifiable through PDF 37-1484. The prominent ZrO_2_ peaks observed in the XRD pattern after the irradiation of ZrN can be attributed to oxidation processes occurring during the laser treatment. These processes are facilitated by irradiation-induced defects, localized heating, and environmental exposure to oxygen, even under the constant argon and nitrogen flow. The fluctuation in peak intensity can be attributed to laser-induced localized heating and subsequent rapid cooling, which trigger processes such as melting and re-crystallization even in the post-deposition treatment stage. With prolonged laser irradiation, the sample progresses through phases of melting, re-solidification, and re-crystallization upon cooling. Gas atoms diffuse along grain boundaries, augmenting the concentration of gas atoms within the sample, thereby manifesting additional peaks or intensifying the peak intensity of the corresponding planes [34,38].

The calculated parameters of all fabricated materials are given in Table 2. The Bragg equation and Debye–Scherrer equations were used to calculate the interplanar spacing (d-spacing) and crystallite size.

The Bragg equation is as follows [49]: nλ=2dsin⁡θ or $d=\frac{n\lambda }{2\sin\theta }$, where *λ* = 1.5406 (wavelength of incident X-rays), n = 1 (order of reflection), *θ* represents the peak position, and *d* indicates the interplanar spacing or d-spacing in (Å). The Debye–Scherrer equation is as follows [50]: $S=\frac{k\lambda }{\beta \cos\theta }$, where *S* represents the crystallite size, *k* represents the Scherrer constant, and *β* is the measured diffraction peak at full-width half-maximum (FWHM). The number of defects in prepared samples was computed by dislocation density (*D*), and calculated using the equation $D=\frac{1}{S^{2}}$ [50,51,52].

**Figure 2:**
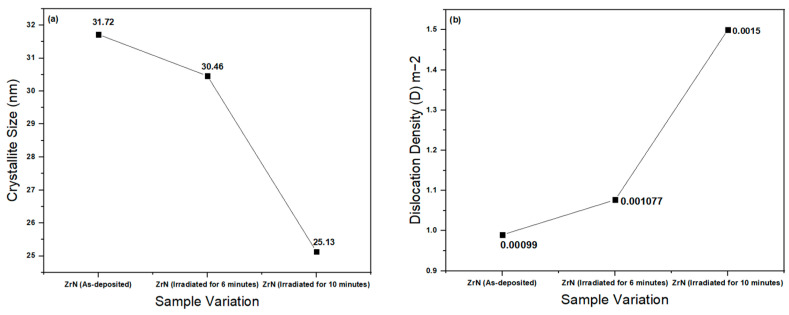
(**a**) Comparison of crystallite size and (**b**) dislocation density of as-deposited/irradiated thin ZrN Film.

Figure 2 presents a comparison of crystallite size and dislocation density of as-deposited and irradiated thin ZrN film. It can be seen in Figure 2a that the crystallite size of the prepared samples [Cubic ZrN (2 0 0)] decreased from the as-deposited state to the irradiated thin ZrN film. The average crystallite size of cubic ZrN (2 0 0) is 29.10 nm. However, the crystallite size of other ZrN peaks when irradiated for 10 min first increased and then decreased rapidly from (2 2 0) to (2 2 2) planes due to the defects [34,40,53] (Table 2). The average crystallite size of all 10 min-irradiated planes of ZrN was 92.21. The initial increase in crystallite size can be attributed to localized heating during irradiation, which promotes recrystallization and grain growth in specific regions. However, as irradiation continues, the generation of defects leads to grain refinement and a reduction in crystallite size. The laser parameters, such as power and duration, can significantly influence the evolution of crystallite size and microstructure. These findings provide valuable insights into the potential structural modifications of ZrN films induced by laser treatment. It can be seen in Figure 2b that the dislocation density of the prepared samples increased from as-deposited to irradiated thin ZrN films. The values of dislocation density show that ZrN produced more defects in the system when irradiated for 10 min [34,51,54,55,56]. The localized annealing and dynamic recrystallizations can increase the crystallinity, along with the dislocation density [57,58]. This can occur under specific irradiation conditions, where energy input is sufficient to reorder the crystalline structure. This dislocation density can also be confirmed with XRD graphs, as co-content has extra peaks, producing more system defects. It is clear from Table 2 that d-spacing of ZrN peaks decreased when irradiated for 10 min. Irradiation can introduce defects that initially reduce the d-spacing by altering the lattice structure. However, it can also lead to annealing effects that repair and reorganize the lattice, resulting in improved crystallinity [59,60]. Results obtained from XRD patterns were in good agreement with the results obtained from SEM and TEM analysis.

### 3.2. Scanning Electron Microscopy (SEM)

**Figure 3:**
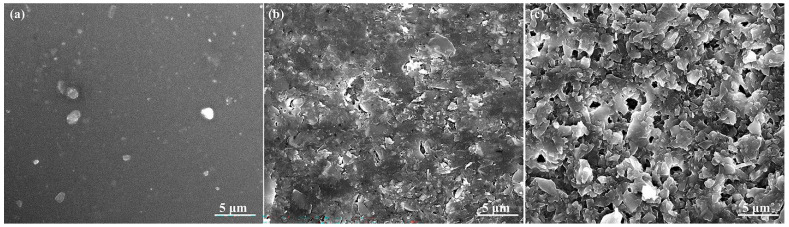
SEM images of as-deposited/irradiated thin ZrN film: (**a**) as deposited; (**b**) irradiated for 6 min; (**c**) irradiated for 10 min.

The investigation delved into the morphology, microstructure, and chemical composition of the deposited layer to comprehend the impact of deposition conditions. Scanning electron microscopy (SEM) images were utilized to scrutinize the top surface of the treated layer, as depicted in Figure 3. The SEM image of the as-deposited ZrN sample in Figure 3a showcases high homogeneity and planarity, with some areas exhibiting small droplets on a smooth surface [61]. Figure 3b illustrates the surface after irradiation for six minutes, displaying regular ablated/melted petals due to laser scanning, leading to surface damage, cracks, and voids within the petals caused by laser irradiation. Upon irradiation for 10 min, as shown in Figure 3c, numerous voids are observed between the petals. Extended laser irradiation enhances the surface mobility of atoms within the film, leading to the formation of defects such as vacancies, interstitials, or dislocations, which in turn promote void formation. This process also contributes to increased hardness of the material. Additionally, irradiation can also induce phase transitions in the material, such as the transformation from cubic ZrN to ZrO_2_, which can result in volume changes that further lead to void formation [42,62,63,64]. In essence, the introduction of oxygen generates voids in the thin ZrN film through irradiation-induced defect formations, oxygen diffusion, oxidation leading to ZrO_2_ formation, volume expansion, and the resulting internal effects that drive void nucleation and growth. The crystallinity of the thin ZrN film was observed to increase post-irradiation.

### 3.3. High-Resolution TEM (HRTEM) Morphology

**Figure 4:**
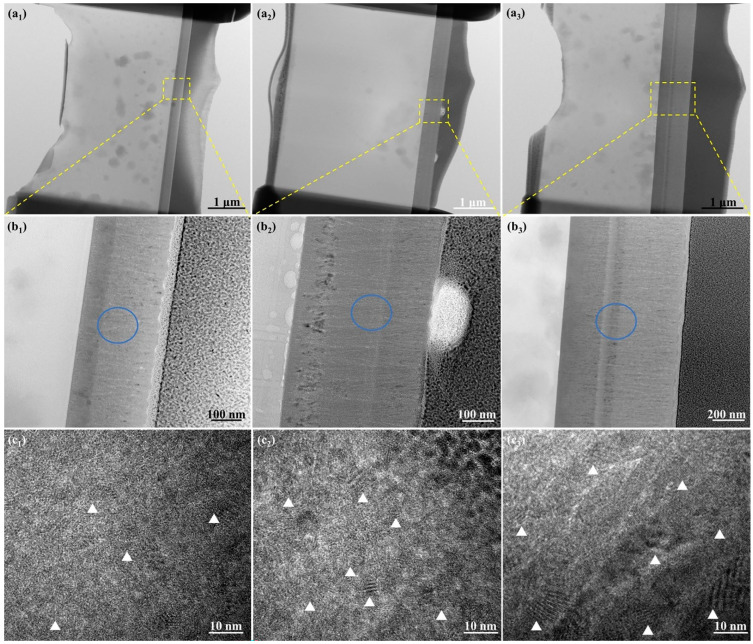
(**a_1_**–**a_3_**) Full TEM views of the foil; (**b_1_**–**b_3_**) magnified views of (**a_1_**–**a_3_**), blue circles indicate the zones, where selected area electron diffraction (SAED) analyses were conducted; (**c_1_**–**c_3_**) bright-field high-resolution TEM (HRTEM) images of the as-deposited 6 and 10 min-irradiated thin ZrN films, triangles indicate the regions exhibiting distinct overlapping grain features.

Figure 4 presents the microstructural evolution of the as-deposited thin ZrN film and those irradiated for 6 and 10 min, denoted a1, a2, and a3, respectively. Selected area electron diffraction (SAED) analyses were conducted at the blue circle zones in b1, b2, and b3. Additionally, c1, c2, and c3 exhibit the bright-field high-resolution TEM (HRTEM) morphology of the as-deposited and 6 and 10 min-irradiated thin ZrN films. The bright-field images reveal uniformly shaped nanoscale grains and features resembling moiré fringes, a consequence of overlapping grains with varying orientations. SAED patterns indicate that the nanocrystalline grains possess random crystallographic orientations. The crystallite size of the surface layer was approximately 20 nm, 15 nm, and 10 nm for the as-deposited and irradiated thin ZrN films, respectively. Notably, the crystallite size observed via TEM was smaller than that computed using XRD, possibly due to the surface nanocrystallized layer being thinner than the detectable depth of XRD measurements [58]. Post-irradiation, the crystallite size of cubic ZrN decreased, generating defects that enhanced the crystallinity of the specimen. Decreased crystallite size correlates with increased hardness of thin films [65], as elucidated by nanoindentation techniques, and a rise in dislocation density. Smaller grains introduce more grain boundaries that impede dislocation movement, bolstering the material’s strength, in line with the Hall–Petch effect [66], where reduced grain size corresponds to heightened hardness. Moreover, heightened dislocation density impedes dislocation motion, enhancing the material’s resistance to deformation. The combination of reduced grain size and increased dislocation density culminated in a robust and resilient thin film.

**Figure 5:**
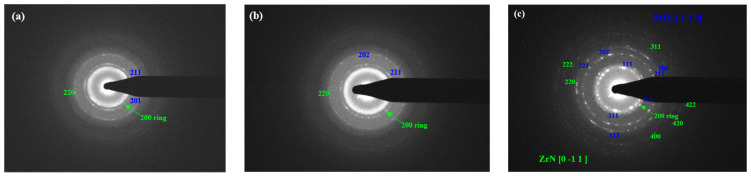
Selected area electron diffraction (SAED) patterns of the blue circled zone in Figure 4(b_1_–b_3_) for as-deposited/irradiated thin ZrN film: (**a**) as deposited; (**b**) irradiated for 6 min; (**c**) irradiated for 10 min. Blue and green show the planes of ZrN and ZrO_2_.

Figure 5 illustrates the selected area electron diffraction (SAED) patterns of the blue circled zones in Figure 4 for both the as-deposited and irradiated thin ZrN films. In these patterns, ZrN and ZrO_2_ planes are represented by green and blue, respectively. The findings reveal a close alignment between the identified planes of ZrN and ZrO_2_ and the results obtained from X-ray diffraction (XRD) analyses. A laser beam’s effects on a coating material depend on the material’s properties, laser parameters, and environmental conditions. The apparent increase in coating thickness in the TEM images could be a result of several factors, including film reorganization or local expansion during the irradiation process. Laser irradiation can induce localized melting or softening of the film, leading to some redistribution of material, which may cause the film to appear thicker in certain areas when observed in cross section.

Laser irradiation has the potential to trigger phase transformations within thin ZrN film. Specifically, under the presence of oxygen, a fraction of the ZrN material might convert to ZrO_2_, either on the surface or within the film. These observations are consistent with EDX data, indicating crystalline structures that differ from the original state, signifying an enhancement in crystallinity following irradiation.

**Figure 6:**
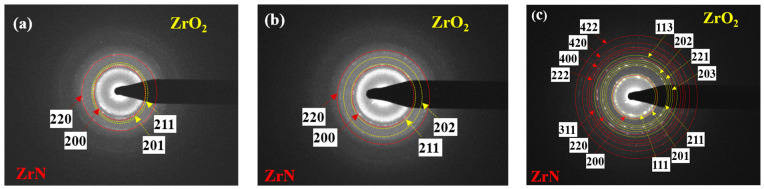
SAED patterns of as-deposited/irradiated thin ZrN film: (**a**) as deposited; (**b**) irradiated for 6 min; (**c**) irradiated for 10 min. Diffraction rings for nanosized ZrN (red) and diffraction rings for nanosized ZrO_2_ (yellow).

Figure 6 showcases the selected area electron diffraction (SAED) patterns of both the as-deposited and irradiated thin ZrN film. In the patterns, diffraction rings for nanosized ZrN are depicted in red, while those for nanosized ZrO_2_ are shown in yellow, indicating the fabrication of nanostructured ZrN. Laser irradiation exhibited a dual role in the thin ZrN film, capable of both generating and healing defects. The high energy of the laser can introduce point defects, dislocations, or voids, while the thermal effects can simultaneously anneal existing defects, thereby reducing dislocation density. In essence, laser irradiation has the potential to significantly modify the microstructure of thin ZrN films through various mechanisms such as phase transformation, grain refinement, defect generation and annealing, alterations in surface morphology, recrystallization, oxidation, and nanoscale structural changes. These effects lead to modifications in grain size, phase composition, defect density, surface characteristics, crystallographic texture, and the presence of oxide layers [5,67].

### 3.4. Dispersive X-Ray Analysis (EDX)

**Figure 7:**
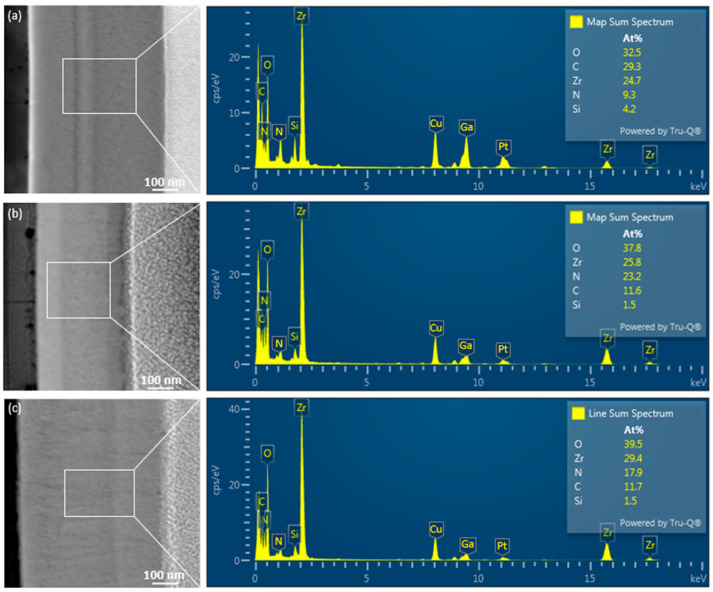
(**a**–**c**) Atomic-scale morphology of the as-deposited and 6 and 10 min-irradiated thin ZrN film captured by AC-TEM along with EDX spectrum.

The dispersive X-ray analysis (EDX) technique provides detailed elemental compositions of materials near the surface of a specimen, along with positional mapping [68]. Figure 7a–c display the atomic-scale morphology of the as-deposited thin ZrN film and those irradiated for 6 and 10 min, captured using aberration-corrected transmission electron microscopy (AC-TEM). The EDX spectrum revealed the presence of zirconium, nitrogen, silicon, oxygen, and carbon in the as-deposited thin ZrN film (Figure 7a). The weight percentages of Zr, N, Si, O, and C were determined to be 24.7%, 9.3%, 4.2%, 32.5%, and 29.3%, respectively. For the 6 min-irradiated ZrN (Figure 7b), the weight percentages were 25.8% Zr, 23.2% N, 1.5% Si, 37.8% O, and 11.6% C, while for the 10 min-irradiated ZrN (Figure 7c), the percentages were 29.4% Zr, 17.9% N, 1.5% Si, 39.5% O, and 11.7% C. The thin film was composed of zirconium nitride, so Zr and N were detected in the EDX spectrum as they were the primary constituents of the film. Oxygen may appear in the EDX results due to the formation of a thin oxide layer (e.g., ZrO_2_) on the surface of the film. Zirconium has a strong affinity for oxygen, and when exposed to air after irradiation, it can react with oxygen to form a native oxide layer. Laser irradiation can introduce defects or induce localized heating, which can enhance the material’s surface reactivity. This may increase the surface energy and allow for the absorption of oxygen from trace amounts in the environment (such as moisture or oxygen adsorbed on the surface), which would explain the increase in oxygen content observed in the EDX analysis. Copper (Cu), gallium (Ga), and platinum (Pt) also appear in the EDX results because a copper grid was used for TEM analysis. Gallium was introduced during FIB preparation, and platinum was deposited as a sputter coating to reduce sample charging and maintain stable electron interaction during TEM/EDX measurements. The EDX results across the entire zones depicted in Figure 7a–c suggest that only C and Si were dissolved in the thin ZrN films. Given that the thin film was deposited on a glass substrate, the presence of silicon in the composition is to be expected. Following sputtering, if the sample is briefly exposed to air, it can adsorb carbon-containing species from the environment. This occurrence is more likely if the sample is not promptly analyzed or stored in a controlled atmosphere, explaining the small amount of carbon detected alongside other elements. Each sample, synthesized at different irradiation times, exhibited distinct elemental compositions.

**Figure 8:**
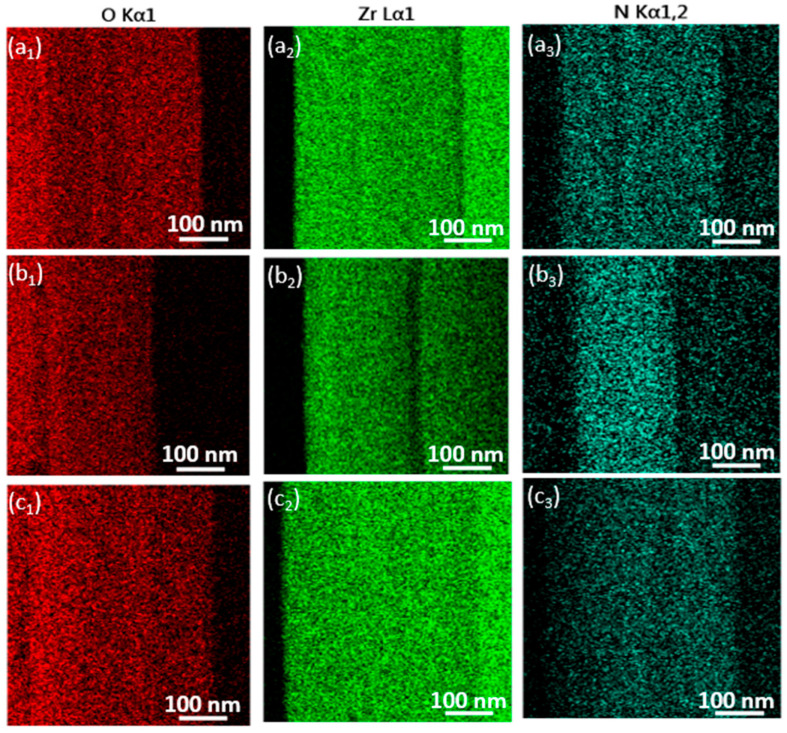
TEM-EDS elemental mapping images of as-deposited/irradiated thin ZrN film: (**a_1_**–**a_3_**) as-deposited; (**b_1_**–**b_3_**) irradiated for 6 min; (**c_1_**–**c_3_**) irradiated for 10 min.

Elemental mapping was conducted on all samples using energy-dispersive X-ray spectroscopy (TEM/EDS). The EDS results for each sample were compared to their respective targeted chemical phases, showing good agreement within the typical error margins of EDS analysis. Figure 8 displays a representative elemental mapping of as-deposited and irradiated thin ZrN film. To confirm the chemical composition, spot EDS measurements were taken on as-deposited and irradiated thin ZrN film, while area EDS was used to assess the homogeneity.

**Figure 9:**
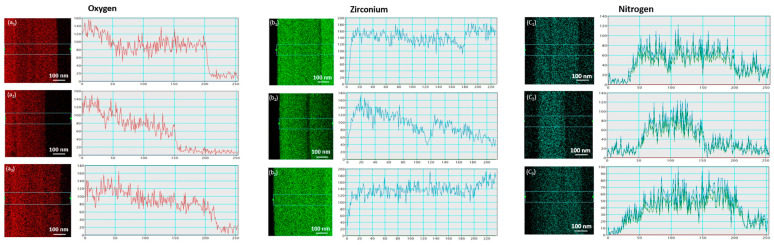
Line EDS of O, Zr, and N: (**a_1_**–**c_1_**) as-deposited, (**a_2_**–**c_2_**) 6 min-irradiated thin ZrN film; (**a_3_**–**c_3_**) 10 min-irradiated thin ZrN film.

Figure 9 illustrates the line-EDS profiles of zirconium (Zr), nitrogen (N), and oxygen (O) for as-deposited and irradiated thin ZrN films. These line scans corroborate the EDX atomic percentage (At. %) data, confirming the elemental distribution within the samples. In the line-EDS maps, the x-axis represents the point number along the scan path, while the y-axis indicates the intensity of each element. The thin ZrN films contained small amounts of C and Si. The presence of Si is due to the glass substrate, while carbon originates from exposure to air. Our analysis focused on ZrN and ZrO_2_, so the line-EDS profiles were studied only for Zr, N, and O. The distinct profiles of Zr, N, and O reflect changes in elemental composition, influenced by the varying irradiation times applied during sample synthesis.

### 3.5. Nanoindentation

**Figure 10:**
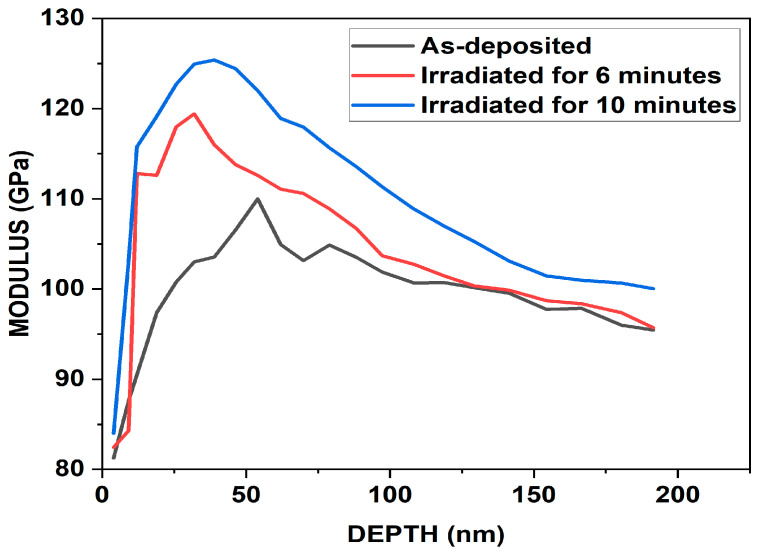
Elastic modulus vs. indentation depth for as-deposited and irradiated ZrN at different times.

**Figure 11:**
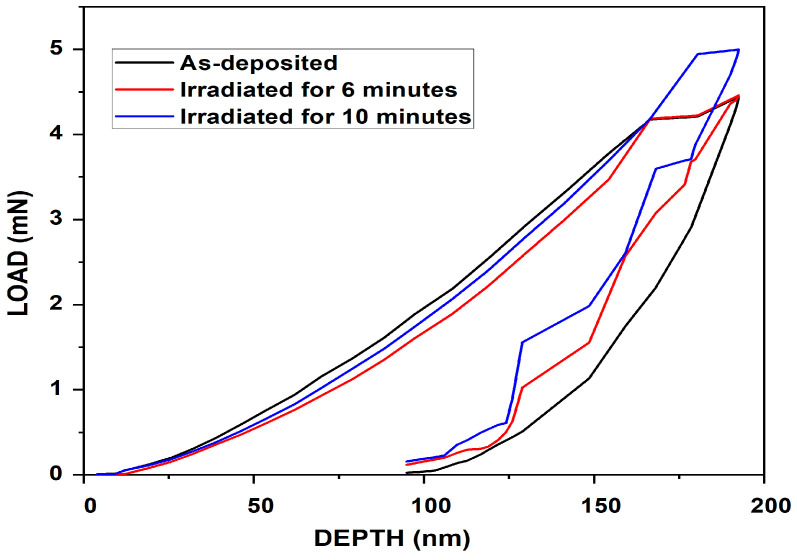
Load vs. indentation depth for as-deposited and irradiated ZrN at different times.

**Figure 12:**
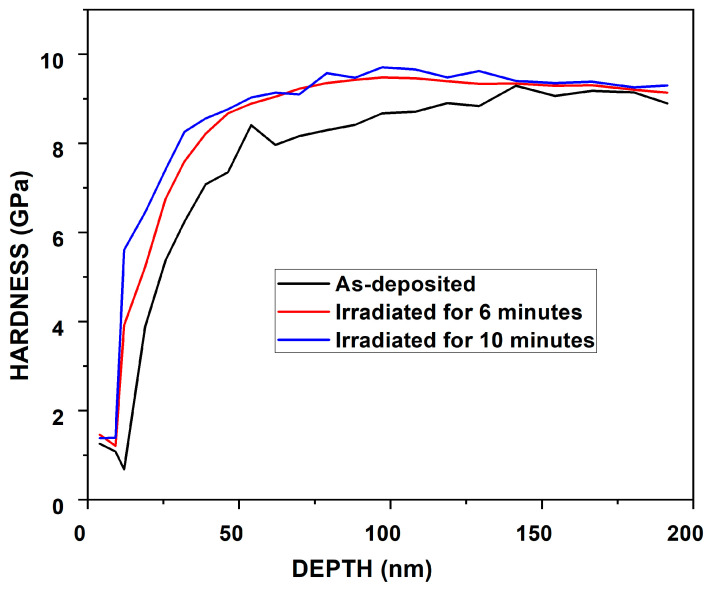
Hardness vs. indentation depth for as-deposited and irradiated ZrN at different times.

The nanoindentation tests were carried out utilizing the continuous stiffness measurement (CSM) indentation technique on a nanoindenter [69,70,71]. The CSM method features depth-controlled indentation, reaching a depth of less than 200 nm. The primary objective of the nanoindentation investigation was to analyze how depth affects the load, elastic modulus, and hardness of the material. Figure 10, Figure 11 and Figure 12 illustrate the moduli of elasticity as a function of indentation depth, utilizing the default settings in the Agilent Nano Indenter G200 and employing the CSM method with a constant strain rate. A notable shift in the modulus of elasticity is evident for primary indentation depths below 150 nm in both the as-deposited and irradiated ZrN samples. This behavior is likely due to surface effects, such as surface oxides, which can influence the material’s mechanical properties and complicate the accurate measurement or estimation of the contact area [72].

Nanoindentation is a widely used technique to evaluate the mechanical properties of thin films. However, it is well established that when the penetration depth exceeds 10% of the film thickness, the substrate significantly influences the measurements. This occurs because the strain field generated during indentation extends beyond the thin film into the substrate, leading to a composite response that reflects both the film and the substrate properties. In our study, while the penetration depths were carefully controlled to remain below the total thickness of the films, we acknowledge that in some cases the 10% rule may have been exceeded. This could have introduced substrate effects, potentially increasing or decreasing the measured hardness depending on the relative mechanical properties of the film and substrate. For example, a harder substrate would elevate the measured hardness, while a softer substrate would reduce it. Similarly, the elastic modulus values could represent a composite response, rather than the intrinsic properties of the thin film. To minimize such effects, we ensured consistent testing parameters, such as maintaining a low maximum load and spacing the indentations to avoid overlapping strain fields.

For the as-deposited ZrN sample, the maximum hardness was observed at a depth of 192 nm. Notably, as the hardness increased, the load and elastic modulus also peaked, as illustrated in Figure 10, Figure 11 and Figure 12. Specifically, in the case of the as-deposited ZrN sample, the maximum hardness, load, and elastic modulus were determined to be 8.91 GPa, 4.44 mN, and 95.46 GPa, respectively. For the ZrN irradiated for 6 min, the maximum hardness, load, and elastic modulus were measured as 9.08 GPa, 4.45 mN, and 95.70 GPa, respectively. Similarly, for the ZrN irradiated for 10 min, the maximum hardness, load, and elastic modulus were found to be 9.36 GPa, 4.99 mN, and 100.03 GPa, respectively.

Following irradiation, lots of defects were created, including dislocations, interstitials, and vacancies. This increase in dislocation density hinders dislocation motion, resulting in enhanced hardness. Moreover, grain size significantly impacts hardness. Smaller grains increase the number of grain boundaries, further impeding dislocation movement and contributing to increased hardness [5,53].

The elastic modulus reflects a material’s stiffness and is linked to the strength of its atomic bonds. Introducing defects such as dislocations or grain boundaries in certain cases creates “local strain fields” that enhance a material’s resistance to deformation. Furthermore, structures with fine grains and higher densities of grain boundaries can enhance stiffness, as these boundaries act as reinforcement points within a material’s structure [73].

In zirconium nitride (ZrN), the elastic modulus may increase due to irradiation-induced defect clusters, nanoscale grain boundaries, and dislocation networks, all of which enhance a material’s mechanical properties, even in the presence of defects.

**Figure 13:**
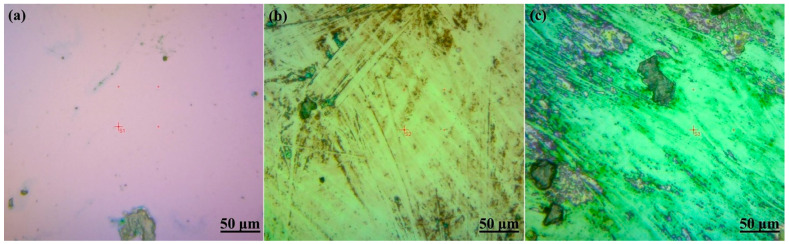
Optical microscopy images: (**a**) as deposited; (**b**) irradiated for 6 min; (**c**) irradiated for 10 min.

Optical microscopy was used to examine the surface and identify any visible cracks or surface features resulting from the CSM method. Figure 13a–c showcase representative optical microscopy images at the maximum indentation depth of 200 nm in the as-deposited and irradiated ZrN samples. In Figure 13a, minimal visible surface cracks or pile-ups are observed. Following irradiation for 6 min, slight alterations can be seen (Figure 13b). However, with the 10-min irradiation period, localized effects led to crack formation, as evidenced in Figure 13c [72].

Upon comparing the results for elastic moduli, load, and hardness, a continuous increase was noted with higher laser irradiation rates, aligning well with the optical microscopy results shown in Figure 13a–c, attributed to the defects generated. Consequently, our findings suggest a hardness enhancement in the prepared thin film.

Specifically, the trend in hardness was as follows: as deposited (8.91 GPa) < irradiated for 6 min (9.08 GPa) < irradiated for 10 min (9.36 GPa).

Hence, the material’s hardness increased with the rise in the laser irradiation time. This occurrence can be ascribed to the ion-exchange reaction occurring under moderate temperature and pressure conditions, acting as a rapid synthesis route for highly crystalline, stoichiometric, and nitrogen-enriched transition metal nitride alloys (TMNAs) [40,74,75].

A noteworthy observation is that the hardness and elastic moduli for the as-deposited ZrN films were lower than the typical values reported in the literature. These discrepancies can be attributed to several factors, including differences in deposition parameters such as substrate temperature, sputtering power, and pressure, which can significantly influence the microstructure of the films. The lower hardness and moduli observed in our as-deposited films may also have resulted from the deposition conditions used, which could have led to lower film quality. Furthermore, oxygen contamination and defects, as indicated by EDX analysis, might have contributed to the reduced mechanical properties of the as-deposited films.

However, following laser irradiation, significant improvements were observed in the mechanical properties of the films, with an increase in both hardness and moduli. This enhancement suggests that the laser irradiation process effectively promotes changes in the microstructure and material properties, likely through mechanisms such as defect healing, crystallization, or phase transformations, resulting in stronger and more resilient films.

### 3.6. Four-Point Probe

**Figure 14:**
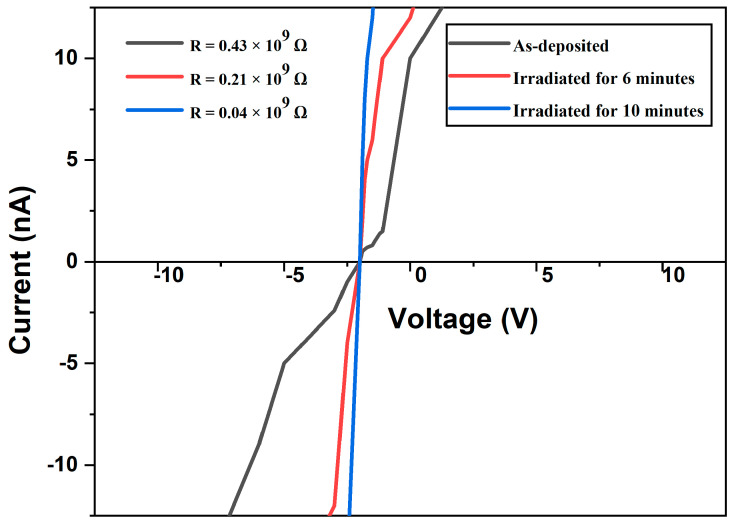
Electrical resistivity of as-deposited and irradiated thin ZrN films.

The electrical resistance of the thin film was assessed using the four-point probe method. Figure 14, illustrates the current–voltage relationships for specific thin ZrN films (as deposited and irradiated for 6 and 10 min) acquired at ambient temperature within an ultrahigh-vacuum environment. The current is plotted on the vertical axis, displaying a direct linear correlation with the applied voltage on the horizontal axis.

Upon analyzing the curves, the sheet resistances of the as-deposited ZrN and the ZrN irradiated for 6 and 10 min were determined to be 0.43 × 10^9^ Ω, 0.21 × 10^9^ Ω, and 0.04 × 10^9^ Ω, respectively. After irradiation, the thin ZrN film exhibited enhanced electrical properties, characterized by a decrease in resistance [76,77,78].

Previous studies have revealed that the resistivity of thin ZrN films is impacted by lattice defects. As the applied bias increases, the film’s resistivity gradually decreases. This decline in resistivity can be ascribed to heightened ion bombardment due to the increased bias, facilitating adatom movement towards their equilibrium positions. Consequently, the film becomes more compact, displaying reduced lattice defects. These alterations contribute to lower resistivity and enhanced crystallinity of the film.

Conversely, the occupation of interstitial positions by nitrogen atoms within the hexagonal close-packed lattice of Zr grain boundaries and lattice distortions results in improved electrical properties of the film [11,79,80,81]. Nevertheless, by adjusting the ZrN ratio, a transition towards the cubic ZrN phase can be induced, effectively reducing the resistivity to levels observed in cubic ZrN [82].

Our findings suggest that extending the irradiation duration decreases the electrical resistance of the thin film, thereby enhancing the electrical properties of ZrN. The availability of such high-quality samples is poised to unlock numerous possibilities in this captivating and burgeoning subfield of materials research.

## 4. Conclusions

In this work, it was demonstrated that laser treatment significantly influences the structural, electrical, and mechanical properties of thin ZrN films. X-ray diffraction (XRD) analysis confirmed the crystalline behavior of the thin ZrN film. Simultaneously, observations from scanning electron microscopy (SEM) analysis revealed the emergence of numerous voids within the film’s surface characteristics due to laser irradiation. High-resolution transmission electron microscopy (HRTEM) examinations unveiled the formation of nanostructured ZrN with identified plane orientations, aligning well with the XRD findings. Additionally, results from the four-point probe measurements indicated that prolonged laser exposure improved the electrical properties of the thin ZrN film, as evidenced by a decrease in sheet resistance from 0.43 × 10^9^ Ω to 0.04 × 10^9^ Ω. Similarly, nanoindentation tests consistently portrayed an uptick in the hardness of the thin ZrN film from 8.91 GPa to 9.36 GPa, highlighting the potential of controlled laser treatment to modify and enhance coating characteristics.

## Figures and Tables

**Table 1:** Deposition parameters of thin ZrN films.

Parameter	Chosen Value
Base pressure	2 × 10^−5^ mbar
Operating pressure	8.5 × 10^−3^ mbar
Substrate-to-target distance	60 mm
Substrate temperature	650 K
Sputtering gas (Ar:N2)	(40:1) sccm
Duty cycle	10%
Sport size	500 µm
Pulse duration	1 µs
Irradiance/laser power density	10 W/cm^2^
Power pulse	19.6 W
Pulse frequency	100 kHz

**Table 2:** Summary of XRD results.

Samples	2θ (Degree)	Crystallite Size (nm)	Dislocation Density (D) m^−2^	d-Spacing (Å)	ZrN (hkl)	Lattice Parameters (Å)
ZrN (As deposited)	40.51	31.72	0.00099	2.29	(2 0 0)	4.5674 (Å) α = 90°, β = 90°, γ = 90°
ZrN (Irradiated for 6 min)	40.51	30.46	0.001077	2.29	(2 0 0)	
ZrN (Irradiated for 10 min)	40.51	25.13	0.0015	2.29	(2 0 0)	
ZrN (Irradiated for 10 min)	55.26	175	0.000326	1.62	(2 2 0)	
ZrN (Irradiated for 10 min)	68.6	158	0.000400	1.36	(3 1 1)	
ZrN (Irradiated for 10 min)	73	133	0.000565	1.32	(2 2 2)	

## Data Availability

The data presented in this study can be obtained from the corresponding author on reasonable request.
